# Pediatric Perspectives on Liver Cirrhosis: Unravelling Clinical Patterns and Therapeutic Challenges

**DOI:** 10.3390/jcm13144275

**Published:** 2024-07-22

**Authors:** Lorenza Forna, Laura Bozomitu, Vasile Valeriu Lupu, Ancuta Lupu, Laura Mihaela Trandafir, Anca Adam Raileanu, Camelia Cojocariu, Carmen Anton, Irina Girleanu, Cristina Maria Muzica

**Affiliations:** 1 Pediatrics—“Sf. Maria” Clinical Emergency Children’s Hospital, 700309 Iași, Romania; lorenza.donea@yahoo.ro (L.F.); valeriulupu@yahoo.com (V.V.L.); anca_ign@yahoo.com (A.L.); trandafirlaura@yahoo.com (L.M.T.); anca.adam60@gmail.com (A.A.R.); 2 Faculty of General Medicine, University of Medicine and Pharmacy “Gr. T. Popa”, 700115 Iași, Romania; cojocariu.salloum@umfiasi.ro (C.C.); carmen.anton@umfiasi.ro (C.A.); irina.girleanu@umfiasi.ro (I.G.); cristina-maria.muzica@umfiasi.ro (C.M.M.); 3 Department of Clinical Gastroenterology, “Sf. Spiridon” Clinical Emergency Hospital, 700111 Iași, Romania

**Keywords:** chronic liver disease, hepatitis B virus, hepatocellular carcinoma, hepatitis C virus, hepatic encephalopathy, PELD, MELD

## Abstract

**Background**: Liver cirrhosis presents significant challenges in the pediatric population due to a complex interplay of etiological factors, clinical manifestations, and limited therapeutic options. The leading contributors to cirrhosis among pediatric patients are chronic cholestasis, metabolic disorders present from birth, and long-term hepatitis. **Materials and method**: Our narrative review aimed to synthesize literature data on the etiology, clinical picture, diagnostic techniques, optimal management of complications, and timely transplantation. **Results**: The epidemiology of liver cirrhosis in pediatric patients is evolving. The introduction of a universal vaccination and effective long-term viral suppression in viral hepatitis have significantly decreased complications rates. Liver transplantation programs worldwide have also improved the management of cirrhosis complications. **Conclusions**: Early diagnosis, comprehensive management strategies, and advancements in treatment modalities are critical for improving outcomes. Understanding these differences is crucial in providing age-appropriate care and support for those affected by cirrhosis.

## 1. Introduction

Liver cirrhosis represents a major challenge in the pediatric population due to a complex interplay of etiological factors, clinical manifestations, and limited therapeutic options. This multifaceted condition, characterized by the irreversible replacement of normal liver tissue with fibrous scar tissue, significantly impacts the pediatric demographic, necessitating a nuanced approach to diagnosis, management, and treatment. The leading contributors to cirrhosis among pediatric patients are chronic cholestasis, metabolic disorders present from birth, and long-term hepatitis.

The prevalence of pediatric liver cirrhosis can vary significantly depending on geographic location, underlying causes, and the availability of healthcare. Globally, the exact prevalence is difficult to pinpoint because liver cirrhosis in children is relatively rare compared to adults [1,2]. However, certain diseases leading to pediatric cirrhosis, like biliary atresia, are among the most common reasons for liver transplantation in children. Biliary atresia alone affects about 1 in every 10,000 to 15,000 live births in the United States [2,3].

Other factors contributing to pediatric liver cirrhosis, such as genetic conditions like alpha-1 antitrypsin deficiency and metabolic disorders like Wilson’s disease, also play a role but have lower incidence rates. The prevalence of these conditions can vary, with alpha-1 antitrypsin deficiency occurring in about 1 in 1500 to 3500 live births in Europeans with around 50 pediatric liver transplants annually due to alpha-1-antitrypsin deficiency, and Wilson’s disease affecting approximately 1 in 30,000 people worldwide [4,5].

Chronic viral hepatitis, a significant cause of cirrhosis, is a major health issue affecting both adults and children, though it is predominantly seen in adults. The prevalence of pediatric cirrhosis due to chronic viral hepatitis would depend on the rates of vertical transmission and early childhood infections in different regions. Cirrhosis has been reported in 1–5% of HBeAg-positive children, while the risk of cirrhosis in children and adolescents with chronic hepatitis C is generally estimated at 1 to 2% [6,7]. Prevalence depends on the geographic region, with Africa and Southeast Asia remaining the areas with the highest number of infected individuals, where the prevalence of HBsAg among children aged up to 5 years reaches 4.7% [6,8,9]. 

Overall, while pediatric liver cirrhosis is uncommon, it is a significant health issue due to its severity and the potential need for liver transplantation [8]. Cirrhosis has long been associated with an incurable disease and a short life expectancy. Now, it is considered a dynamic condition, which can be reversed if adequately treated. According to studies from the Brazilian Unified Health System, between 2001 and 2010, liver diseases were the eighth leading cause of death in Brazil. Cirrhosis was the main cause of hospital admissions and death from liver disease, especially in the South region of the country [1]. Little is known about its incidence in children.

The aim of this narrative review was to focus on the main aspects of the etiology, pathophysiology, diagnosis, and management of liver cirrhosis.

## 2. Materials and Methods

The search strategy included a comprehensive review of the literature using the Web of Science and PubMed databases. Keywords such as “pediatric cirrhosis”, “chronic liver disease”, “liver transplantation”, and “management of liver complications” were utilized. We focused on retrospective and prospective studies published in the last 10 years. Inclusion criteria were studies that provided data on pediatric patients, with a preference for those offering insights into the latest advancements in diagnostic and therapeutic strategies. The literature search was performed in March 2023, ensuring the inclusion of the most recent and relevant studies.

## 3. Results and Discussion

Pediatric liver cirrhosis arises from a diverse array of causes, with congenital and genetic disorders, metabolic diseases, and chronic viral infections being predominant. The etiological diversity (Table 1) underscores the importance of a comprehensive diagnostic evaluation to tailor interventions effectively.

In a study from Iran with 106 pediatric patients, the most common causes of liver cirrhosis were Wilson’s disease *(n* = 22; 20.7%), biliary atresia (*n* = 19; 17.9%), and cryptogenic cirrhosis (*n* = 14; 13.2%); none of the patients had liver cirrhosis caused by an infection. The most frequent complications of liver cirrhosis in children in the study were ascites (44.3%), gastrointestinal variceal bleeding (16.1%), and hepatic encephalopathy (12.7%), and jaundice (67.9%), which is not actually a complication but has been observed during the exacerbation of the disease. Adults, on the other hand, often develop cirrhosis due to hepatitis B, B+D, C infection, chronic alcohol abuse, and NAFLD (non-alcoholic fatty liver disease), with complications that can include encephalopathy and hepatocarcinoma [10]. 

In Japan, among children who received liver transplants due to cirrhosis, the predominant causes were identified as biliary atresia, accounting for 72.9% of cases, followed by unidentified origins (cryptogenic) at 8.1%, Budd–Chiari syndrome and progressive familial intrahepatic cholestasis, each contributing to 5.4% of the instances, and Wilson’s disease, representing 2.7% of the cases [11]. 

In addition to family history, other risk factors for chronic liver disease include the trend among teenagers towards tattoos and piercings, which are associated with hepatitis C, as well as the use of alcohol or other drugs [11]. It is also worth noting the connection with other associated diseases such as inflammatory bowel diseases leading to sclerosing cholangitis, hemolytic anemias, jaundice, and endocrine abnormalities [6,11].

In the early months of 2022, instances of severe hepatitis in young children, not linked to typical viruses, were reported in Scotland, followed by similar reports from the USA and other parts of the UK. This surge coincided with an increase in COVID-19 cases among children, attributed to the Omicron variant, leading to speculation that SARS-CoV-2 might be responsible for this unusual disease. Later research pointed towards adenovirus type 41, species F (Ad-F41), alone or in combination with adeno-associated virus 2, as a more likely cause [12]. With up to 80% of patients showing signs of Ad-F41 infection, this became the primary suspect, even though the specific pathological markers of adenoviral hepatitis were not always present. While the exact cause of the outbreak remains unclear, this situation highlights the potential for viral infections to trigger severe liver conditions in children, contributing to our understanding of factors that might also influence pediatric cirrhosis [12]. 

### 3.1. Clinical Manifestations

Cirrhosis is a condition marked by widespread fibrosis and nodular regeneration, resulting in the disruption of normal liver structure [13,14]. Liver cirrhosis represents the final stage of chronic liver disease, characterized by the cycle of hepatocyte necrosis and regeneration that leads to fibrosis and the capillarization of hepatic sinusoids. This condition is also associated with an increased risk of developing hepatocellular carcinoma. For instance, in Japan, the primary cause of liver cirrhosis is hepatitis C virus (HCV) infection. Studies of the natural history of cirrhosis have found that the disease tends to present with a silent clinical course, followed by the onset of liver dysfunction and portal hypertension [15,16,17] (Table 2). The most important predictor of decompensation is the increase in hepatic venous pressure gradient (HVPG), which is seldom measured routinely in children and adolescents [7,16].

When registering a child, aspects related to the mother and pregnancy are considered, as well as the infant’s clinical condition, birth weight, APGAR score, vaccination, jaundice and its duration, any surgical interventions, procedures, or other associated conditions [7].

The clinical presentation of hepatic cirrhosis in children can be insidiously progressive, making early diagnosis challenging. Initial symptoms may be non-specific, such as failure to thrive, fatigue, maybe nausea, vomiting, or diarrhea. As cirrhosis advances, more pronounced signs emerge, including jaundice, pruritus, portal hypertension, abdominal distention, ascites, splenomegaly, and variceal hemorrhage. The progression to decompensated cirrhosis is marked by severe complications like hepatic encephalopathy and hepatorenal syndrome, which significantly impact morbidity and mortality rates [7,14,15].

Complications arising from cirrhosis encompass a range of serious conditions such as jaundice, ascites, gastrointestinal variceal bleeding, and hepatic encephalopathy, all of which signal a progression to a more severe, decompensated stage of the disease. Additionally, complications can include spontaneous bacterial peritonitis, edema, hepatopulmonary syndrome and hepatorenal syndrome [10].

Rajan et al. described an unusual case in Singapore of Hepatocellular Carcinoma (HCC) in a patient who was negative for Hepatitis B e Antigen (HBeAg), exhibited a low viral load, and showed no biochemical or histological signs of active hepatitis, liver fibrosis, or cirrhosis [18]. The patient was born to an HBV carrier mother. He received HBV immunoglobulin at birth and the full course of HBV vaccine but had no follow-up until 9 years of age when blood tests showed positive for HBsAg. In the moment of diagnosis had a raised value of Alpha-fetoprotein—53,606 μg/L. This confirms the unpredictability of the progression of liver diseases, in this case, the infection with hepatitis B virus, which, as it turns out, rapidly progressed to hepatocellular carcinoma without going through the preceding stages of fibrosis or cirrhosis. Therefore, regular monitoring is crucial, even for asymptomatic patients [18]. Current guidelines may not mandate routine HCC screening in the absence of cirrhosis among hepatitis B patients, but this case prompts reconsideration of monitoring practices, especially in pediatric contexts with similar risk profiles [19]. 

At the physical examination can be noticed:

Episodes of hepatic encephalopathy (HE) can be triggered by a range of factors, including sepsis, bleeding in the upper gastrointestinal tract, dehydration, constipation, ileus, or small bowel obstruction, among others [20]. The severity of HE may be intensified by various mechanisms such as oxidative stress, states of high inflammation, changes in cerebral blood flow, and metabolic disturbances. The exact cause of HE remains unclear, with several theories under consideration. A prominent theory suggests that gut-derived toxins and elevated ammonia levels play a key role, but alternative explanations involving changes in bile acids, cytokines, and chemokines have also been proposed [7,20]. 

Hepatic encephalopathy (HE) associated with acute liver failure is classified as Type A HE (Table 3). Conversely, patients experiencing HE due to portosystemic shunting without any underlying liver disease fall under the classification of Type B HE. Type C HE is identified in individuals with chronic liver disease, such as cirrhosis or portal hypertension, accompanied by HE. Within Type C HE, there are various subtypes, including covert, persistent, and episodic forms of the condition (Table 3).

The standard grading of pediatric hepatic encephalopathy (HE) typically utilizes the West Haven criteria, despite being originally developed based on adult data. These criteria have been adapted to suit the pediatric population. In children, the manifestations of HE can be less distinct, marked by subtle changes in mental status that still indicate progressive encephalopathic alterations [22]. These signs in pediatric HE range from irritability and fatigue to confusion, and in severe cases, can escalate to coma (Table 4) [23,24].

### 3.2. Diagnostic Approaches

Early detection and characterization of liver cirrhosis are mandatory for effective management. The diagnostic algorithm integrates clinical evaluation, laboratory tests (including liver function tests, coagulation profiles, and specific markers of liver damage), imaging studies (ultrasound with Doppler, MRI, and transient elastography), and liver biopsy. Genetic testing and metabolic studies are essential for diagnosing underlying etiologies in cases suspected to have a genetic or metabolic basis [24].

As a first non-invasive test, abdominal ultrasonography can reveal a nodular liver surface and ascitic fluid in Morison’s pouch. The severity and nature of morphological changes in liver cirrhosis are influenced by the disease’s progression and specific type. In the early stages of compensated cirrhosis, a common finding among many patients is an enlarged liver [8]. Early manifestations also include the dilation of the porta hepatis, the expansion of interlobar fissures, and the increase in the size of the space surrounding the gallbladder. With disease advancement, there is a notable increase in fibrosis and a decrease in the liver’s total volume, that can be accompanied by capsular retraction [13]. Commonly observed is a contrast between lobar hypotrophy and the hypertrophy of the left lateral lobe, which causes the liver to extend towards the left. In cases of left lateral lobe hypertrophy, there’s a noted “kissing” interaction with the spleen. Ultrasound also observes the liver’s texture, providing a qualitative assessment of the liver tissue. While a healthy liver displays a consistent and even texture on ultrasound, fibrosis-affected liver tissue exhibits increased echogenicity, characterized by irregular distribution among adjacent pixels and enhanced beam attenuation [14]. 

Transient elastography (Fibroscan) is a non-invasive method for investigating hepatic fibrosis, easy to perform and with rapid results. It is conducted using an ultrasonic transducer probe that produces vibrations of low amplitude and frequency. The speed of wave propagation through the liver tissue is directly proportional to the stiffness of the parenchyma, so the more fibrotic the tissue, the faster the wave propagates. Its applicability is influenced by the presence of ascites or significant adipose tissue which can lead to inconclusive results. In such cases, MRE (Magnetic Resonance Elastography) is often preferred [25,26].

Elastography values in patients with chronic HBV infection, for example, are closely correlated with the stages of the infection. In a recent study in 125 patients with inactive HBV infection, the average value was 4.8 ± 1.2 kPa, in contrast to patients with active disease where the value of significant fibrosis (≥F2) is in the range of 5.2 ± 8.7 kPa. An F4 grade of fibrosis implies the presence of cirrhosis, and cases without complications have values between 10.3 and 13.4 kPa [27].

Moreover, in Wilson’s disease, metabolic disorders (such as Gaucher’s or Pompe disease), congenital heart defects, cystic fibrosis, or steatohepatitis, elastography plays an important role in detecting hepatic fibrosis, thus protecting the pediatric patient from the invasiveness of liver biopsy [28]. 

The METAVIR scoring system, used in transient elastography to assess liver fibrosis, categorizes the extent of fibrosis on a scale from F0 to F4 based on the elasticity measurements in kilopascals (kPa). An F0 score, indicating no fibrosis, is characterized by values less than 6.1 kPa. Mild fibrosis, or F1, ranges from 6.1 to 6.9 kPa. Moderate fibrosis, classified as F2, spans from 6.9 to 7.5 kPa. Severe fibrosis, or F3, is identified with values ranging from 7.5 to 14.1 kPa. Finally, F4, indicating cirrhosis, is defined by values greater than 14.1 kPa. This scoring system helps in quantifying the degree of liver stiffness and therefore the severity of fibrosis, guiding clinical management and intervention strategies [14,25,29]. 

### 3.3. Biochemical Tests

Several biochemical markers can be utilized to help diagnose and monitor the progression of liver cirrhosis. Here are some examples:

- Complete Blood Count (CBC)—Useful for detecting signs of infection, inflammation, and anemia, which can be secondary to liver disease.
- Biochemical Tests: ALT (Alanine Aminotransferase) and AST (Aspartate Aminotransferase), also known as serum aminotransferases or transaminases, are highly sensitive indicators of liver damage. ALT is more specific to the liver, primarily found in hepatocytes, making it a more specific marker of liver health than AST, which is also present in muscles, kidneys, brain, pancreas, and lungs [30].
- Bilirubin and Its Fractions: Increased levels can indicate liver dysfunction. Its fractions (direct and indirect) help determine the type of jaundice.
- Alkaline Phosphatase (ALP), Gamma-Glutamyl Transferase (GGT): Elevated levels can indicate cholestasis or damage to the bile ducts.
- Ammonia: Often correlating with hepatic encephalopathy.
- Total Proteins and Albumin: Decreased levels in chronic liver disease due to the liver’s reduced synthetic capability.
- Cholesterol (Total and Fractions) and Triglycerides: Lipid profile changes can occur in liver diseases affecting metabolism.
- Iron (Fe), Ferritin, and Transferrin: If Hemochromatosis is suspected.
- Copper (Cu), Ceruloplasmin and 24 h urinary copper (for children older than 3 years): Important in the diagnosis of Wilson’s disease [30].
- Coagulation profile—PT (Prothrombin Time), INR (International Normalized Ratio), and APTT (Activated Partial Thromboplastin Time) [31].
- α1-Antitrypsin: This protein’s levels are measured to diagnose α1-antitrypsin deficiency.
- Sweat Test: Primarily used for diagnosing cystic fibrosis, which can lead to liver dysfunction as a secondary complication.
- Viral markers: HBsAg, HDV Ag, Anti-HCV Antibodies.
- Alpha-fetoprotein (AFP)—is considered a marker of disease progression towards hepatocellular carcinoma, especially when it exceeds values of 400 ng/mL. It can also be elevated in episodes of exacerbation with significant cytolysis [32,33].
- Smooth Muscle Antibodies (SMA), Mitochondrial Antibodies (AMA), Anti-Nuclear Antibodies (ANA), Anti-Liver/Kidney Microsome Type 1 Antibodies (Anti-LKM-1)—if suspected autoimmune hepatitis, primary biliary cholangitis.
- HIV serology
- Genetic tests: Pompe disease, Gaucher disease, Alagille syndrome, etc. [1].

Laboratory tests are crucial for evaluating liver function, detecting hypersplenism, and identifying the underlying causes of liver disease. In cases of obstructive liver damage, levels of canalicular enzymes, such as alkaline phosphatase and gamma-glutamyl transferase (GGT), along with bilirubin levels, tend to be elevated. However, these enzymes do not reflect hepatic synthesis and lack prognostic significance [34].

Hepatocellular carcinoma (HCC) is observed in patients with chronic liver disease and can occur in children although it is rare in viral pathology, being more common in metabolic and cholestatic etiologies. During physical or ultrasound examinations, an abdominal mass may be detected, at which point alpha-fetoprotein levels may be elevated. An Italian study found that HCC was present in 2% of 103 children with cirrhosis who underwent primary liver transplantation [32]. Despite the early onset age, the prognosis after liver transplantation was excellent and there was no recurrence of the disease. Children with cirrhosis of any etiology should undergo abdominal ultrasound examinations and measurements of alpha-fetoprotein every 6 months or at least once a year [32]. Alpha-fetoprotein (AFP) is considered a marker of disease progression towards hepatocarcinoma, especially when it exceeds values of 400 ng/mL. It can also be elevated in episodes of exacerbation with significant cytolytic activity [33].

In cases of significantly high ammonia levels, studies have highlighted an increased risk of cerebral herniation when these levels exceed 200 μmol/L. Elevated ammonia in the bloodstream creates an osmotic gradient, drawing water into brain cells. This osmotic shift can cause brain cells to swell, leading to increased intracranial pressure. Over time, this increased pressure can result in cerebral herniation, a life-threatening condition where parts of the brain are pushed through openings in the rigid structure of the skull [34]. The presence of hypoalbuminemia and coagulation factor deficiencies are closely linked with diminished hepatic synthesis and offer better predictions of survival outcomes [1]. These markers are superior compared to other clinical or liver function tests because they directly reflect the liver’s synthetic capacity (EASL 2021). Coagulation tests and hypoalbuminemia are essential for assessing the severity and prognosis of chronic liver disease or cirrhosis. Albumin levels reveal the liver’s synthetic capacity and offer insights into nutritional and overall health status, while coagulation tests act as prognostic indicators. Conversely, ALT and AST are primarily utilized for diagnosing and monitoring liver inflammation (EASL 2021). Commonly used in settings where patients are evaluated for liver transplantation or managing complications of liver disease, these indicators are integral to scoring systems such as the Child-Pugh score and the MELD score. They help assess disease severity and predict survival, focusing on the likelihood of surviving liver-related complications or overall mortality [34]. 

The assessment of liver disease severity is conducted using the Child-Pugh classification system, the “Pediatric End-stage Liver Disease (PELD)” scoring system for individuals younger than 12 years, and the “Model for End-stage Liver Disease (MELD)” scoring system for those aged 12 years and older (Table 5) [35].

The PELD score = 0.480 × ln (bilirubin in mg/dL) + 1.857 × ln (INR) − 0.687 × ln (albumin in g/dL) + 0.436, if the patient is under 1 year old [35].

PELD = 0.480 × ln (bilirubin in mg/dL) + 1.857 × ln (INR) − 0.687 × ln (albumin in g/dL) + 0.667, if the patient has growth failure (>2 SD—defined as height or weight > 2 standard deviations below the mean for age). The resulting sum is multiplied by 10 and rounded, with the value being directly proportional to the risk of death. The PELD score is used for children up to 12 years old and was introduced into medical practice in 2002 by the Organ Procurement and Transplantation Network (OPTN) in the United States, aiming to reflect the pre-transplant prognosis and prioritize patients. The PELD score is specifically designed to estimate the risk of mortality within a 90-day period [35,36]. The score is used to prioritize liver transplantation in pediatric patients, with higher scores indicating a greater risk of death within this timeframe without a transplant However, studies indicate that the PELD may underestimate the severity of the condition of candidates on the liver transplant waiting list, suggesting a need for revising the prognostic algorithm in pediatric patients. Chang and colleagues, in a study of 4298 children, over 50% of whom were under 1 year old, found that the accuracy of the PELD score does not match that of the MELD score, especially at high values and young ages. The variability in the risk of death within 90 days at a PELD > 30 ranged between 23% and 43%. The mortality risk associated with the MELD score is presented in Table 6 [36].

The MELD score is calculated as 0.957 × ln (creatinine in mg/dL) + 0.378 × ln (bilirubin in mg/dL) + 1.120 × ln (INR) + 0.643. The resulting sum is multiplied by 10 and rounded, with scores ranging from 6 to 40 [37] (Table 5).

**Table 5:** The interpretation of MELD score values (modified after Kamath et al., 2001) [37].

MELD Score	Re-Evaluation
<10	At 1 year
11–18	At the 3 months
19–24	At 1 month
>25	Every week

The use of the MELD score in patients with advanced liver disease aged ≥12 years underwent a modification in 2016, such that individuals with a score of ≥12 will also have their serum sodium value included [38]. The formula is updated as follows:

MELD-Na score, an adaptation of the original MELD score that incorporates serum sodium levels to improve the prediction of mortality in patients with end-stage liver disease. This adjustment recognizes the additional risk posed by hyponatremia (low sodium levels) in liver disease patients [38].

MELD-Na = MELD(i) + 1.32 × (137 − Na) − [0.033 × MELD(i) × (137 − Na)], where MELD (i) is the original MELD score calculated from bilirubin, INR, and creatinine, and Na is serum sodium levels, measured in mEq/L.

**Table 6:** The risk of mortality based on the MELD score values (modified after Biggins et al.) [38].

MELD Score	Mortality Risk
40	71.3%
30–39	52.6%
20–29	19.6%
10–19	6.0%
≤9	1.9%

The Child-Pugh scoring system, also known as the Child-Pugh-Turcotte score, was originally developed by Child and Turcotte in 1964. It was intended to predict mortality among cirrhosis patients and to help select candidates for elective portal decompression surgery. The scoring system was later refined by Pugh and colleagues, who replaced the measure of clinical nutrition status with prothrombin time to enhance its accuracy. The score is calculated based on 5 parameters: total bilirubin, albumin, prothrombin time, encephalopathy, and ascites, each being scored with 1, 2, or 3. The total score is the sum of these points, from 5 to 15. Thus, there are 3 Child-Pugh classes: A (5–6 points), B (7–9 points), and C (10–15 points) [39].

Serum biomarkers for hepatic fibrosis typically involve a combination of various blood tests, used together to enhance the accuracy of assessments (EASL 2021 [40]. The two most well-known indices, which are based on commonly available laboratory markers, are the aspartate aminotransferase (AST) to platelet ratio index (APRI) and the Forns index, although caution is necessary if APRI is used in patients from Africa, as its sensitivity is reduced for this group [40]. Other tests include those that test for specific biochemical markers related to fibrinolysis or fibrinogenesis [41].

FibroTest (BioPredictive, Paris, France) and FibroSure (LabCorp, Burlington, NC, USA) test for gamma-glutamyltransferase (GGT), total bilirubin, alpha2-macroglobulin, apolipoprotein A1, and haptoglobin [42]. They have been validated for use in patients with CHB (Chronic Hepatitis B) and are widely used. Other tests or non-invasive serum measurements specific for fibrosis include FibroMeter (Echosens, Paris, France) and the Enhanced Liver Fibrosis (ELF) score [43].

### 3.4. Therapeutic Challenges

The management of pediatric hepatic cirrhosis is tailored to the underlying cause, the extent of liver damage, and the presence of complications. Therapeutic strategies encompass nutritional support, pharmacotherapy to address specific etiologies (e.g., ursodeoxycholic acid for cholestatic liver diseases, immunosuppressants for autoimmune hepatitis), and interventions to manage complications (such as endoscopic variceal ligation and beta-blockers for portal hypertension). Liver transplantation remains the definitive treatment for end-stage liver disease, offering the potential for a significantly improved quality of life and survival. However, challenges such as donor availability, surgical risks, and post-transplantation care, including lifelong immunosuppression, pose significant considerations.

**Compensated cirrhosis** often presents without symptoms until it progresses to a more severe stage with complications. However, the progression from CLC to decompensated cirrhosis involves complex interactions among various predisposing and precipitating factors. The occurrence of the first decompensation event marks a critical juncture in the disease’s progression, indicating a sharp drop in median survival rates from 10–12 years to just 1–2 years [23].

To manage this condition, several strategies are recommended:

- Preventing additional liver damage by avoiding substances toxic to the liver;
- Conducting screening endoscopies to check for varices; if found, employing medical and endoscopic treatments as a preventive measure against variceal bleeding (Non-selective beta-blockers (NSBBs), such as propranolol or nadolol, are commonly used. Endoscopic Treatment: Endoscopic variceal ligation (EVL) is employed if NSBBs are contraindicated or not tolerated by the patient);
- Regularly (every 6 months) screening for hepatocellular carcinoma [23].

**Decompensated cirrhosis** signals its presence through the development of ascites, non-obstructive jaundice, bleeding from gastrointestinal varices, and hepatic encephalopathy. These symptoms significantly elevate the risk of mortality. 

To manage decompensated cirrhosis, the following strategies are employed:

- Managing ascites through sodium restriction and the use of diuretics, with large-volume therapeutic paracentesis for cases unresponsive to medical treatment; a shunting procedure might be required for severe cases [44,45,46];
- Regular endoscopic monitoring for varices, along with prophylactic endoscopic banding for those at high risk of bleeding (Patients without varices may be monitored every 2–3 years/Patients with small varices might be monitored more frequently, such as annually, to detect progression to larger varices that might warrant prophylactic treatment). High risk factors include the size of the varices, the presence of red wale markings and the severity of liver dysfunction [46].
- Treating hepatic encephalopathy with lactulose and rifaximin to reduce symptoms;
- Conducting regular screenings for hepatocellular carcinoma [23];
- Also, patients with liver cirrhosis should avoid of drugs that can harm the liver, such as NSAIDs in any amount and acetaminophen in doses exceeding 2 g per day. Beta-blockers are recommended for preventing variceal bleeding, but their use is appropriate only within a certain timeframe. This period starts when moderate to large esophageal varices appear and ends once refractory ascites or hypotension (defined as a mean arterial pressure of 82 mm Hg or lower) occur [17]. Surgery within the abdomen, other than for liver transplantation, should be avoided for those with decompensated cirrhosis unless the potential benefits significantly outweigh the risks [45]. Specifically, cholecystectomy poses a particularly high risk of increased morbidity and mortality in these patients [46,47].
- **Lactulose**, a semisynthetic disaccharide composed of β-galactosidofructose, serves as a cornerstone in the management of hepatic encephalopathy. It facilitates the conversion of ammonia into ammonium (NH4+), which is subsequently trapped in the stool. This mechanism effectively reduces the amount of ammonia available for absorption. The pediatric dosage for lactulose is 0.3–0.4 mL/kg, administered two or three times per day [48].
- **Lactitol**, a nonabsorbable disaccharide, operates in the same manner as lactulose, aiming to reduce blood ammonia levels. Its primary benefit lies in its powdered form, making it less sweet and more convenient for use compared to lactulose [49].
- **Neomycin**, an oral antibiotic, has been effectively utilized in treating early hepatic encephalopathy in children. For adults, the recommended dosage is between 4–6 g per day. In pediatric cases, a starting dose of 1 g is advised [50].
- **Liver dialysis**, specifically through systems like the Molecular Adsorbent Recirculating System (MARS), is being explored for its potential to improve outcomes in hepatic encephalopathy by removing toxins from the blood. Its effectiveness and precise role, however, are still under investigation, with its use mainly confined to research settings [22].

### 3.5. Liver Transplantation

Pediatric Liver Transplantation (PLT) stands as a critical treatment for children facing end-stage organ failure, significantly impacting not only the patient but their entire family. In 2022, the United States saw 9528 liver transplants, with 6143 more conducted up until 2023, according to OPTN data [51,52]. Advances in surgery, anesthesia, and intensive care have notably improved long-term survival rates following transplantation. Undergoing liver transplantation can lead to profound alterations in the lifestyles of both the children undergoing the procedure and their parents, affecting family dynamics and functions. These changes, including variations in physical appearance like weight gain or stunted growth, can harm the child’s body image and self-esteem. Additionally, negative perceptions from peers can challenge these children’s abilities to form and sustain friendships, leading to feelings of alienation, decreased social belonging, and increased anxiety among pediatric transplant recipients [51]. 

Organ rejection is a critical concern following transplantation, introducing uncertainty about the future, fears of mortality, and heightened anxiety for pediatric patients [53]. The confrontation with a life-threatening condition and the subsequent adjustment to living with a chronic illness often lead to maladaptive behaviours among children, such as tantrums, impulsivity, aggression, or inattentiveness. It’s widely recognized that children living with chronic conditions or those who have undergone transplantation face significant risks for mental health issues and challenges in psychosocial adaptation [54]. A study was undertaken by M.E. Düken and E.H. Yayan to assess the levels of trait anxiety, depression, and post-traumatic stress symptoms in children who have received liver transplants, highlighting the psychological impact of organ transplantation and the importance of addressing these mental health concerns. The results indicate that three years after surgery, 47.9% of children exhibit severe post-traumatic stress and 43.7% experience moderate post-traumatic stress following transplantation [55].

#### 3.5.1. Indications for Pediatric Liver Transplantation (modified after Vimalesvaran et al.) [55]

- Decompensated Chronic Liver Disease and Its Complications: This includes conditions like refractory ascites, encephalopathy, spontaneous bacterial peritonitis, hepatopulmonary syndrome, or hepatorenal syndrome;
- Acute Liver Failure Meeting Transplant Criteria;
- Portal Hypertension Refractory to Medical and Endoscopic Therapy;
- Refractory Pruritus;
- Recurrent Cholangitis in Cholangiopathies;
- Severe Growth Failure in Chronic Liver Disease: Despite optimal nutritional management, severe growth failure occurs;
- Unresectable Liver Tumors;
- Liver-based Metabolic Defects: Conditions which can be cured or significantly improved by transplantation.

#### 3.5.2. Contraindications for Pediatric Liver Transplantation (modified after Vimalesvaran et al.) [55]

- Overwhelming Active Extrahepatic Infections;
- Extrahepatic Malignancy;
- Severe Uncorrectable Cardiopulmonary Disease;
- Severe Neurological Injury;
- Multi-systemic Progressive Disorders: Disorders that affect multiple systems of the body, such as mitochondrial diseases, which show progressive deterioration.

End-stage liver disease is marked by severe complications that indicate the liver’s inability to perform its essential functions. These complications include synthetic failure, evidenced by prolonged prothrombin time and low albumin levels in the blood, and difficulty in managing variceal bleeds, where hemostasis is not achievable despite multiple endoscopies or requires additional interventions like the Sengstaken–Blakemore tube. Other signs include persistent ascites, inadequate nutrition despite targeted dietary efforts, hepatopulmonary syndrome, and hepatic osteodystrophy. However, the outlook post transplantation is positive, with a survival rate exceeding 80% at 20 years [56]. 

In pediatric care, several surgical techniques for liver transplantation have been developed to accommodate different needs and situations. The most common methods include whole liver transplant, split-organ grafts, reduced organ grafts, and auxiliary liver transplant. Each technique has its unique approach and application depending on the patient’s specific condition and the availability of donor organs [56]. 

The complications that can arise after liver transplantation, emphasizing the importance of monitoring and managing these potential issues across different stages post-transplant are listed below in Table 7 (Note: The early post-operative period refers to the time immediately following the surgery up to around 6 months post-transplant. The late post-operative period starts after the early post-operative phase, from 6 months onward, extending into the years following the transplantation) [55].

### 3.6. Nutrition in Chronic Liver Disease

Liver disease disrupts the normal processes of appetite, digestion, absorption, assimilation, storage, and metabolism of nutrients, both macro and micronutrients. For infants with liver disease, breastfeeding is recommended to continue unless there is a specific underlying metabolic issue that necessitates cessation. Parenteral nutrition is preferred only when oral or nasogastric feeding is not feasible. In cases where feeding by mouth presents challenges, the European Society for Clinical Nutrition and Metabolism (ESPEN) endorses the use of nasogastric (NG) feeding. NG tubes, particularly those made from polyurethane or silicone, are generally well-received and pose a minimal risk of causing bleeding. These can be used for either bolus feeds throughout the day or continuous overnight feeding [57]. 

Malnutrition exacerbates liver disease, while liver cirrhosis, in turn, negatively impacts nutritional status, creating a harmful cycle that must be addressed early with nutritional intervention. Carbohydrates should constitute 40–60% of the total energy intake [58]. To address the issues of negative nitrogen balance and the protein requirements for growth, infants need a protein supplementation of 3–6 g/kg/day, while children require 2 g/kg/day. MCTs (medium-chain triglycerides) are advantageous because they can be absorbed without bile acids, offering greater bioavailability. In cases of chronic liver disease (CLD), it is recommended that MCT oil comprises about 30–60% of the total dietary fat. For infants, various formula feeds enriched with MCTs are available and can serve as supplementary feeds following breastfeeding. Additionally, MCT oil can be administered separately at a total daily dosage of 1–2 mL/kg, divided into 2–4 doses [59,60]. 

Sodium intake should be limited to 1.5 to 2 mEq/kg/day since salt can lead to water retention, exacerbating conditions like edema and ascites. Medications, including diuretics, can significantly affect the balance of electrolytes, such as sodium and potassium. Therefore, it is important for children taking these medications to undergo regular blood tests to monitor their electrolyte levels [61]. 

## 4. Conclusions

Pediatric hepatic cirrhosis demands a multidisciplinary approach to address its complex clinical patterns and therapeutic challenges. Early diagnosis, comprehensive management strategies, and advancements in treatment modalities are critical for improving outcomes. Continued research and innovation in pediatric hepatology will pave the way for more effective and targeted therapies, enhancing the quality of life and survival prospects for affected children. The impact of cirrhosis on children versus adults showcases the complexity of this disease and its broad spectrum of effects. Children face unique challenges related to growth, development, and nutrition, while adults contend with complications that can significantly affect life expectancy and quality of life. Understanding these differences is crucial in providing age-appropriate care and support for those affected by cirrhosis.

## Figures and Tables

**Table 1:** Conditions potentially leading to cirrhosis in children and adolescents (modified after Pinto et al., 2015) [1].

Conditions	Specific Cause
Biliary Obstruction Causes	Biliary atresia
	Choledochal cysts
	Gallstones
	Bile duct stenosis
Familial intrahepatic cholestasis	Alagille syndrome
	FIC1 deficiency (ATP8B1)
	BSEP deficiency (ABCB11)
	MDR3 deficiency (ABCB4)
	Defects in bile acid synthesis
Hepatotropic Viral Infections	Hepatitis B and D
	Hepatitis C
Inherited Genetic–Metabolic Diseases	Alpha-1-antitrypsin deficiency
	Glycogen storage diseases type III and IV
	Galactosemia
	Fructosemia
	Tyrosinemia type 1
	Wilson’s disease
	Mitochondrial hepatopathies
	Late cutaneous porphyria
	Cystic fibrosis
	Hemochromatosis
	Wolman disease
	Drugs and Toxins
Drugs and Toxins	Total parenteral nutrition
	Isoniazid
	Methotrexate
	Vitamin A intoxication
Autoimmune Diseases	Autoimmune hepatitis
	Primary sclerosing cholangitis
	Vascular Alterations
Vascular Alterations	Budd–Chiari syndrome
	Veno-occlusive disease
	Congenital cardiopathy
	Congestive heart failure
	Constrictive pericarditis
Other Causes	Fatty liver disease
	Neonatal hepatitis
	Zellweger syndrome

**Table 2:** Clinical aspects of cirrhosis in children (Modified after Pinto et al., 2015) [1].

Clinical Aspects of Cirrhosis in Children	
General Appearance	anthropometric measurements to identify malnutrition/obesity, fever
Skin and Limbs	jaundice, skin color changes such as flushing or pallor, presence of spider angiomas, telangiectasias, redness of the palms, nail clubbing, xanthomas, and Terry’s nails
Abdominal Examination	abdominal distension, visible blood vessels, changes in liver and spleen size (including decreased liver volume and spleen enlargement)
Neurological Assessment	evaluates academic achievements, sleep patterns, presence of asterixis, Babinski sign, alterations in mental status
Additional Findings	delayed puberty, gynecomastia, shrinkage of the testicles, and signs of feminization

**Table 3:** Classification of HE phenotypes (Modified after Bartlett et al., 2024) [21].

Type	Description
Type A	Encephalopathy associated with acute liver failure.
Type B	Encephalopathy due to portosystemic bypass without intrinsic hepatocellular disease.
Type C	Encephalopathy associated with cirrhosis.
Type D	Encephalopathy associated with disorders of the urea cycle.

**Table 4:** Grading of Pediatric Hepatic Encephalopathy Using West Haven Criteria [21,23].

HE Grade	Clinical Features (Younger Children, <4 years)	Clinical Features (Older Children, >4 years)
Grade 1	Increased fussiness, sleep disturbance, easily distracted	Sleep disturbance, mood changes, mental fogginess
Grade 2	Increased fussiness, sleep disturbance, easily distracted	Progressive fatigue
Grade 3	Somnolence, agitation	Increased fatigue, confusion, speech abnormalities, hyperreflexia, and increased tone
Grade 4 (coma)	Overt coma, decerebrate or decorticate posturing	Overt coma

**Table 7:** Complications following liver transplantation and the importance of monitoring and management at various post-transplant stages adapted from Vimalesvaran, S. et al. [55].

Complications of PLT	Time Frame	Type
Surgical complications	Early post-operative period	Surgical
Primary graft dysfunction	Early post-operative period	Surgical
Hepatic artery thrombosis	Early post-operative period	Surgical
Portal vein stenosis and thrombosis	Early post-operative period	Surgical
Hepatic vein outflow obstruction	Early post-operative period	Surgical
Biliary leaks and stenosis	Early post-operative period	Surgical
Acute rejection	Early post-operative period	Medical
Infection	Early post-operative period	Medical
Late liver allograft dysfunction	Late post-operative period	Medical
Late-onset acute rejection	Late post-operative period	Medical
Chronic rejection	Late post-operative period	Medical
Idiopathic post-transplant hepatitis	Late post-operative period	Medical
Recurrence of liver disease	Late post-operative period	Medical
Post-transplant lymphoproliferative disorder	Late post-operative period	Medical
Long-term biopsychosocial outcomes	Long term	Biopsychosocial
